# Differential Functions of C- and N-Terminal Hepatitis B x Protein in Liver Cells Treated with Doxorubicin in Normoxic or Hypoxic Condition

**DOI:** 10.1371/journal.pone.0050118

**Published:** 2012-11-29

**Authors:** Davor Kin-fan Chau, George Gong Chen, Haitao Zhang, Billy Cheuk Sing Leung, Sukying Chun, Paul Bo-san Lai

**Affiliations:** Department of Surgery, The Chinese University of Hong Kong, Prince of Wales Hospital, Shatin, N. T., Hong Kong, China; University of North Carolina School of Medicine, United States of America

## Abstract

Hepatitis viral B x protein (HBx), a hepatocarcinogen, is frequently mutated. Hypoxia influences the growth of HCC and also the sensitivity of tumor cells to treatments. We aimed to test the role of HBx and acute hypoxia in the efficacy of chemotherapy. In this study, we established 4 Chang liver cell lines with the full-length HBx (HBx), the first 50 amino acids of N-terminal HBx (HBx/50), the last 104 amino acids of C-terminal HBx (HBx/51) and empty vector (CL), respectively. MTT and TNUEL assays were used to assess cell viability and apoptosis respectively. Western blot was used to determine the expression of relevant proteins. Results showed that among 4 cell lines, doxorubicin was most effective in decreasing the viability and enhancing apoptosis in HBx/51 cells, while HBx/50 cells were most resistant to the treatment. Cells in hypoxia were more susceptible to doxorubicin than cells in normoxia. Hypoxia facilitated the Bid cleavage especially in HBx/51 cells via phosphorylating p38 MAPK. p38 MAPK inhibitor significantly reduced the tBid level and increased cell viability. In conclusion, N-terminal HBx and C-terminal HBx function differentially in their ability to regulate cell growth, with the former being promotive but the latter being inhibitory. The acute hypoxia may overcome the HBx-induced resistance and facilitate the chemotherapy.

## Introduction

Hepatitis B virus X protein (HBx), a major product of hepatitis B virus (HBV), is well known to implicate in hepatocarcinogenesis [1], [2]. HBx can affect a range of cellular events related to cell proliferation and growth. Interestingly, in term of apoptotic regulation, HBx showed dual functions, promotion and inhibition. For example, HBx may activate Notch signaling or upregulate SATB1 expression to inhibit the apoptosis in HCC cells [3], [4]. On the other hand, HBx is shown to enhance apoptosis induction via degrading Mcl-1 and activating TNF-receptor 1 [5], [6]. The reason for the dual function of HBx is not entirely known at present though it may relate to HBx mutants, the length/duration of the infection and types of cells.

Hypoxia participates in the development of cancer as well as the cancer treatment. It can exert different effects on the growth of cancer cells. Usually, the chronic hypoxia is in favor of hepatocarcinogenesis and metastasis and also renders cancer cells resistant to chemotherapy [7], [8]. In contrast, the acute or transient hypoxia may sensitize HCC cells to anti-tumor treatments. For example, under the hypoxic condition, the cytotoxic effect of chemotherapeutic agent doxorubicin can be enhanced [9], [10]. The mechanism responsible for the hypoxia-induced sensitivity to anti-tumor agents is not completely known. However, the acute or transient hypoxia can render cells more susceptible to apoptosis [11], [12]. Among various molecules affected by hypoxia, Bid was found to be cleaved under hypoxia [13], [14]. In addition to hypoxia, doxorubicin may also activate Bid [15], [16].

Bid, a pro-apoptotic molecule, participates in both intrinsic and extrinsic pathways. Traditionally, Bid is cleaved by caspase 8 to generate the truncated Bid (tBid), a more potent pro-apoptotic molecule. tBid will activate the oligomerization of Bax and Bak in mitochondria and subsequently lead to a series of downstream apoptotic events such as the release of cytochrome c and the formation of the complex apoptosome [17], [18]. Though both hypoxia and doxorubicin may induce Bid cleavage [13]–[16], it is unknown how HBx may impact hypoxia- and/or doxorubicin-induced Bid cleavage in liver cells. Considering the fact that HBx possesses a dual function in the regulation of apoptosis, this question remains particularly interesting. In this study, we attempted to answer this question by establishing liver cells that expressed the full-length HBx, C-terminal HBx and N-terminal HBx and determining how these cells responded to doxorubicin in normoxic and hypoxic conditions.

## Materials and Methods

### Generation of HBx and mutant HBx plasmids and the corresponding stable cell lines

Wild-type full-length HBx, the fragment containing the first 50 amino acids (a.a.) (1–50), and the fragment containing 51–154 a.a. were constructed basically according to previous description [19]. Briefly, the fragments were respectively amplified from full length HBx (accession no: DQ448619) by PCR and cloned into pcDNA3.1 (Invitogen, Carlsbad, CA). PCR was performed with Expand High Fidelity^PLUS^ PCR System (Roche, Mannheim, Germany) using the below primers, in which an EcoRI restriction site and a NotI restriction site were incorporated into the forward and reverse primers respectively. The sequences of the primers used were as follows: HBx: Forward primer: 5′-CCGA.A.TTCA.A.CCATGGCTGCTAGGCTGTGC, Reverse primer: 5′-GA.A.TGCGGCCGCATTAGGCAGAGGTGA.A.A.A.AGT; HBx/50: Forward primer: 5′-CCGA.A.TTCA.A.CCATGGCTGCTAGGCTGTGC, Reverse primer: 5′-GA.A.TGCGGCCGCTTACCCGTGGTCGGTCGGTAC; HBx/51: Forward primer: 5′-CCGA.A.TTCACTATGGCGCACCTCTCTTTACGCG, Reverse primer: 5′-GA.A.TGCGGCCGCATTAGGCAGAGGTGA.A.A.A.AGT. The PCR products were digested by EcoRI and NotI and cloned into pcDNA3.1. The sequence of the successful clone was confirmed by DNA sequencing.

Human normal hepatic Chang Liver (CL) cells were obtained from Type Culture Collection of Chinese Academy of Sciences (www.cellbank.org.cn) and transfected with the relevant plasmid DNAs and the stable cell lines were established by the G418 (400 µg/ml) (Calbiochem, San Diego, CA) selection for 3 weeks. Four stable cell lines were established: HBx (cells transfected with the full-length HBx gene), HBx/50 (cells transfected with the first 50 a.a. of HBx), HBx/51 (cells transfected with the sequence of 51 to 154 a.a. of HBx), CL (cells transfected with the empty vector pcDNA3.1). The expression of the relevant HBx in the established cell lines was verified by RT-PCR (Fig. S1). The cells were cultured in a complete medium containing Dulbecco's Modified Eagle's Medium, supplemented with 10% heat inactivated fetal bovine serum and 1% Penicillin/Streptomycin. The medium was changed every 2 days until the cells being 90% confluent.

### Generation of hypoxic conditions

Hypoxic condition (0.1% O_2_) was generated by incubating cells in a hypoxic chamber (Galaxy R, RS Biotech, Germany) with 95% N_2_, 0.1% O_2_, 4.9% CO_2_ at 37°C for the different periods detailed in the result section or relevant figures.

### Cell culture and cell viability assay

Cell viability was assessed by MTT assay [18]. The loss of viable cells can represent the cytotoxicity induced by anticancer agents. To confirm the result of MTT assay, we used a non-isotopic immunoassay for quantification of BrdU incorporation into newly synthesized DNA of actively proliferating cells. Briefly, cells were seeded in a 96-well microtitre plate. Doxorubicin and/or hypoxia was applied to cells to generate stress. At 4 h before the end-point of stress-treatment, the working solution of BrdU was incorporated into the proliferating cells. The cell culture continued for additional 4 h either in hypoxia or normoxia. At the end of stress treatment, the cell proliferation was determined by the CHEMICON®'s BrdU Cell Proliferation Assay Kit. The plate was read with a wavelength of 450 nm, with a reference of 595 nm. The greater optical density (OD) of the sample, the higher the BrdU concentration in the sample.

### Western blot analysis

Western blot was performed according to the previous publication [18]. The antibodies for human hypoxia-inducible factor 1alpha (HIF-1α), p53, actin, Bid and tBid were purchased from Santa Cruz Biotechnology (Santa Cruz, CA); Bax, Akt, phosphor-Akt, p38 MAPK and phosphor-p38 MAPK antibodies were obtained from Cell Signalling (Danvers, MA); and HBx was provided by Applied Bioreagents (Golden, CO).

### TUNEL assay

APO-DIRECT™ TUNEL Assay kit (Chemicon, San Diego, CA) was used to detect the apoptosis. Cells were treated with doxorubicin and incubated under hypoxic or normoxic condition. After treatment, the medium was collected and the adherent cells were washed. The floating cells in the medium were collected after centrifugation. The adherent cells were trypsinized by trypsin-EDTA, and collected in the tube that contained the floating cell pellet. The cells were then centrifuged to remove the supernatant. The rest of steps was performed according to the instruction of the manufacturer.

### p38 MAPK

Cells were trypsinized and seeded in a 6-well plate at 3×10^5^ cells per well. 24 h prior to doxorubicin treatment, p38 MAPK inhibitor SB203580 was added into the corresponding samples. After the cells were incubated for 24 h, doxorubicin was added. The doxorubicin-treated cells were incubated in hypoxia or normoxia for another 24 h. Cells were then harvested to determine the expression of phospho-p38 MAPK, p38 MAPK and Bid by Western blot. The cell proliferation was determined by performing MTT assay as described above.

### Statistical analysis

The data presented as the mean ± SD for at least three independent determinations for each group. The differences between the groups were examined for statistical significance using one-way analysis of variance and/or Student's t test. *P*<0.05 was considered as significant.

## Results

### Verification of hypoxia

**Figure 1:**
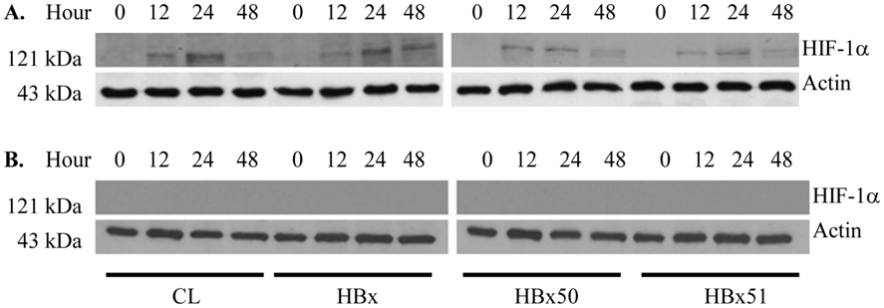
Expression of HIF-1α in cells under hypoxia (A) and normoxia (B). In the hypoxic condition, cells were cultured in 0.1% oxygen. The total protein was isolated from the cells that were cultured at hypoxic or normoxic condition for the different periods as indicated in the figure. Western blot was performed to determine the expression of HIF-1α. Actin protein was served as a loading control.

To verify the state of hypoxia generated in this study, we determined HIF-1α expression, since it is regarded as an indicator of hypoxia [7], [8]. HIF-1α will undergo proteosomal degradation in normoxia but it is stable in oxygen deficient environment. Fig. 1 showed that HIF-1α was detected in all four established cell lines when the cells were incubated in hypoxia but not in normoxia.

### Cell viability and apoptosis

**Figure 2:**
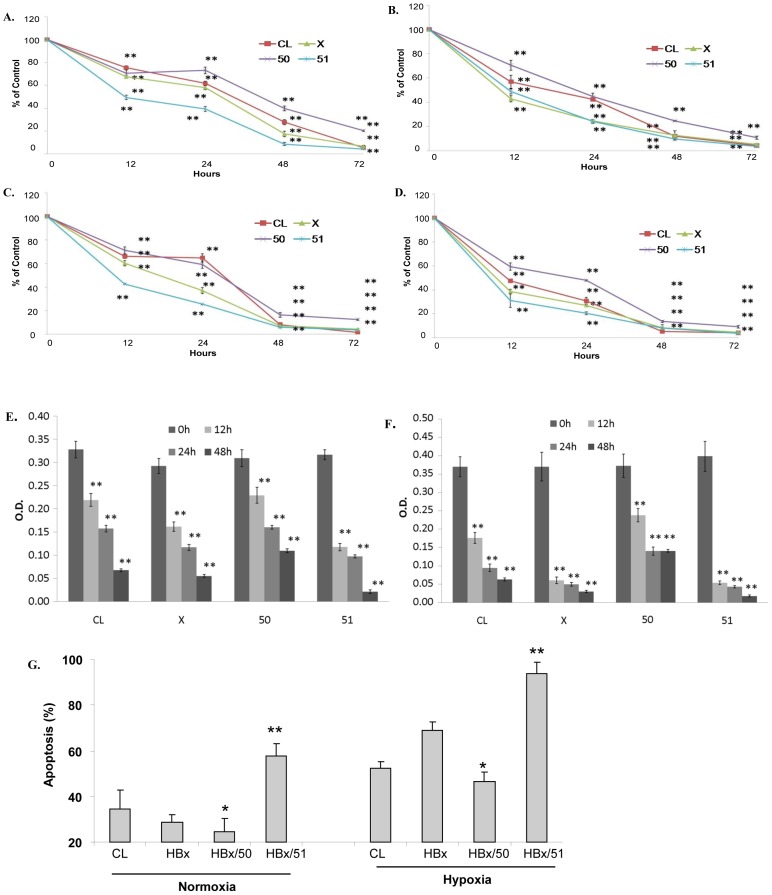
Response of cells to stress stimulation. Cells were treated with 2 µM or 5 µM doxorubicin under normoxic or hypoxic condition for different periods as indicated in the figure. At the end of the treatment, the viability of each cell lines was determined by MTT assay (**A–D**). **A** and **B**: cells were treated with 2 µM doxorubicin in the normoxic (**A**) or hypoxic (**B**) condition. **C** and **D**: cells were treated with 5 µM doxorubicin in the normoxic (**C**) or hypoxic (**D**) condition. Cell viability was calculated using the formula: absorbance of the particular cell at testing period/absorbance of the particular cell at time 0(control). The results are presented in mean±SD calculated from at least 9 independent experiments. In addition to MTT assay, cell proliferation was determined using BrdU incorporation method. **E:** cells were cultured under the hypoxic condition and treated with 2 µM doxorubicin. **F**: cells were cultured under the hypoxic condition and treated with 5 µM doxorubicin. The OD values of **E** and **F** reflect the concentration of proliferating cells in a direct proportion manner and the results are presented in mean±SD calculated from 9 independent experiments. **p<0.001 when compared with the control (0 point). **G:** Cells were cultured under the normoxic or hypoxic condition and treated with 2 µM doxorubicin for 48 h and apoptotic cells were determined by TUNEL assay. *p<0.05, **p<0.001 when compared with CL.

In order to test the response of CL, HBx, HBx/50 and HBx/51 to stress stimulation, cell viability was measured after the cells were treated with 2 or 5 µM doxorubicin for different periods as indicated in figures. The result showed that doxorubicin was able to significantly reduce cell viability or cause cytotoxicity in all 4 cell lines tested in both normoxic and hypoxic conditions (Fig. 2). Among the 4 cell lines, HBx/50 cells that contain the N-terminus of HBx was most resistant to doxorubicin in both normoxic and hypoxic conditions, whereas HBx/51 cells that have the C-terminus of HBx was most sensitive to the treatment (Fig. 2A–2D), indicating that C- and N-terminal HBx fragments may differentially affect the cell response to stress stimulation. The viability of HBx cells was lower in most points than that of CL cells. However, the difference appeared to be insignificant. Under the same concentration of doxorubicin and the same duration of treatment, all four stable cell lines had less viable cells under hypoxia (Fig. 2B and 2D) than under normoxia (Fig. 2A and 2C), suggesting that hypoxia may facilitate the doxorubicin-induced damage. For example, the percentages of cell viability for CL, HBx cells, HBx/50 and HBx/51 treated with 2 µM doxorubicin and under the normaxia were 69±2%, 61±3%, 77±4% and 42±3% respectively (Fig. 2A); whereas the percentages of cell viability for these 4 cell lines treated with 2 µM doxorubicin and under the hypoxia were 46±2%, 29±2%, 53±3% and 24±2% respectively (Fig. 2B). The reduction in cell viability was more obvious in cells treated with the high dose of doxorubicin (5 µM, Fig. 2C and 2D) compared with those treated with the lower dose of doxorubicin (2 µM, Fig. 2A and 2B). In the absence of doxorubicin, HBx/51 cells were also less viable than HBx/50 cells (data not shown). The result of the cell viability (cytotoxicity) measured by MTT assay was confirmed by the BrdU cell proliferation assay (Fig. 2E and 2F). TUNEL assay demonstrated that doxorubicin induced apoptosis in all cells tested (Fig. 2G). However, the percentage of apoptotic cells was much higher in cells under hypoxia than cells under normoxia and HBx/51 cells were most sensitive to the treatment among 4 cell lines tested.

### Expression of apoptotic proteins

**Figure 3:**
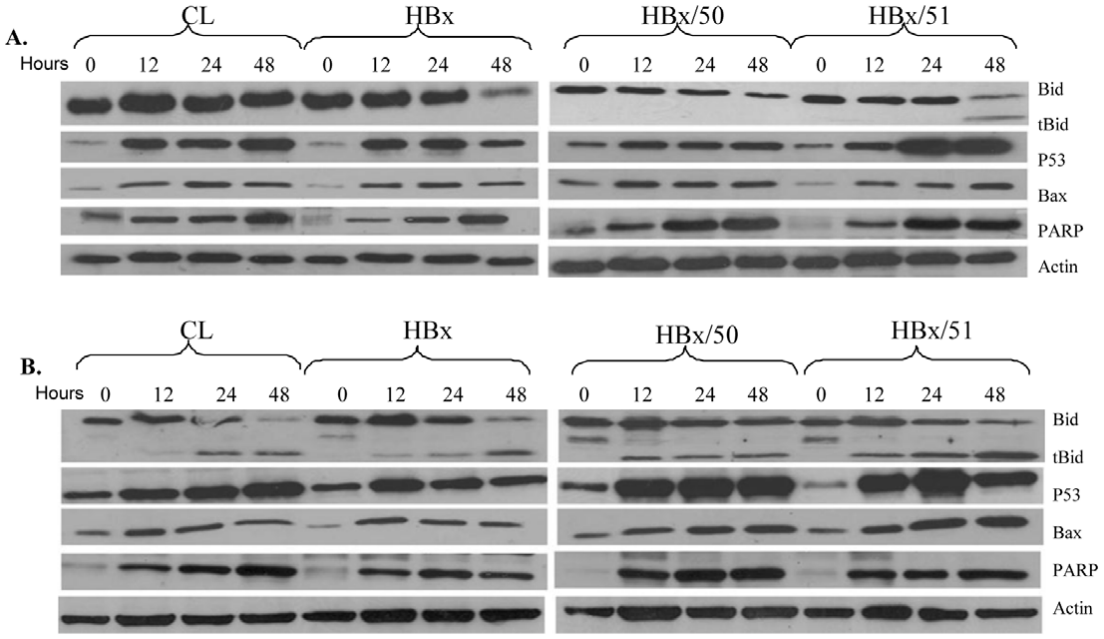
Expression profiles of apoptotic proteins. Cells were cultured in the normoxic (**A**) or hypoxic (**B**) condition, and treated with 2 µM doxorubicin. At the end of treatments, total protein was isolated and subjected to Western blot for Bid, tBid, p53, and Bax. Actin was used as a loading control.

To investigate the possible mechanism responsible for the effects of oxygen conditions and doxorubicin on cells, the expression profiles of the apoptotic proteins were analyzed. When the cells were treated with doxorubicin under the normoxic condition, the levels of the pro-apoptotic proteins Bid, p53, and Bax in all four cell lines were increased (Fig. 3A). tBid, which is formed from the cleavage of Bid, was shown in some cells such as HBx/51 (Fig. 3A). In order to investigate how hypoxia enhanced the effect of doxorubicin, a parallel experiment was performed in which the cells were incubated in the hypoxic condition and the levels of apoptotic proteins were analyzed by Western blot. It was found that changes in p53, and Bax were similar to that seen in the normoxic counterparts when the cells were treated with the same concentrations of doxorubicin (Fig. 3B). However, unlike in the normoxic condition, tBid was detected in all four cell lines in the hypoxic condition, and the highest level of tBid was found in HBx/51 cells. It was noted that there was an additional band below Bid in untreated cells showed in Fig. 3B. The reason of this is unclear but they may be non-specific bands which somehow disappear after the treatment. Nevertheless, further experiments need to clarify it.

### p38 MAPK in the induction of tBid

**Figure 4:**
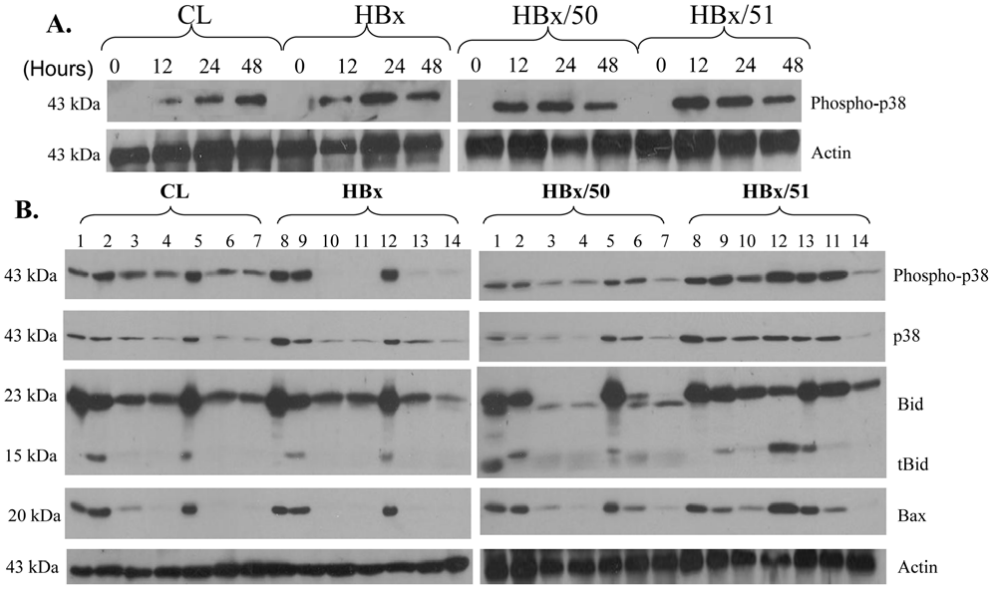
Expression of p38 MAPK. **A:** Cells were treated with 5 µM doxorubicin for 48 h under the hypoxic condition. At the end of treatments, total protein was isolated and subjected to Western blot for phosphor-p38 MAPK. Actin was used as a loading control. **B:** To confirm the role of p38, cells were cultured in either normoxic or hypoxic condition and treated with 2 µM doxorubicin in the presence or absence of 50 µM SB203580 for 48 h. At the end of treatments, total protein was isolated and subjected to Western blot for phospoho-p38, Bid, tBid, and Bax. Actin was used as a loading control. Lanes 1 and 8: Control under normoxia; Lanes 2 and 9: treated with 5 µM doxorubicin under normoxia without SB203580; Lanes 3 and 10: treated with 5 µM doxorubicin under normoxia with 50 µM SB203580; Lanes 4 and 11: Control under hypoxia; Lanes 5 and 12: treated with 5 µM doxorubicin under hypoxia; Lanes 6 and 13: treated with 5 µM doxorubicin under hypoxia with 50 µM SB203580; Lanes 7 and 14: treated with 50 µM SB203580.

Since p38 MAPK and Akt have been reported to be associated with Bid activation [20]–[22], the expression of Akt and p38 MAPK was investigated by immunoblotting. It was found that the level of the phosphorylated p38 MAPK was detected from 12 h after the addition of doxorubicin in all four cell lines in the hypoxic condition (Fig. 4A). More importantly, the phosphor-p38 MAPK was synchronized with the detection of tBid (Fig. 4B). Doxorubicin treatment did not induce the phosphorylated Akt in the hypoxic condition (data not shown). Taken together, these results imply that the activation of p38 MAPK but not Akt is likely to participate in the cleavage of Bid in the cells tested.

To investigate whether the cleavage of Bid could be inhibited by blocking the activation of p38 MAPK, the cells were pre-treated with the p38 MAPK inhibitor SB 203580 for 24 h followed by doxorubicin for 48 h. It showed that SB203580 significantly inhibited the level of phosphorylated p38 MAPK (Fig. 4B). SB203580 was the most effective in blocking the phosphorylated p38 MAPK in HBx cells that contained the full length HBx. tBid was induced by doxorubicin in all cells tested, particularly in the HBx/51 cultured in hypoxia (Lanes 2, 5, 9, and 12, Fig. 4B). Importantly, when the phosphorylated p38 MAPK was inhibited by SB203580, the corresponding tBid bands were significantly downregulated (Lane 14, Fig. 4B) or even disappeared (Lanes 3, 6, and 10, Fig. 4B), suggesting that phosphorylation of p38MAPK may be responsible for the Bid activation. It should be noted that SB203580 could not 100% block doxorubicin-induced tBid in HBx/51 cultured in hypoxia but it was able to do so in other three types of cells. These findings once again support that HBx/51 is most sensitive to doxorubicin among the cells tested.

## Discussion

Two HBx mutants, HBx/50 and HBx/51, were constructed and used in this study. HBx/50 is a N-terminal deletion HBx and contains the first 50 a.a. whereas HBx/51 is a C-terminal deletion HBx and contains the last 104 a.a. It is known that the first 50 a.a. in N-terminal HBx is the region for transactivation repression and the C-terminal region between 51 and 154 contains the domain for the transactivation [19], [23]. Furthermore, HBx mutants with the C-terminal deletion are frequently reported in HCC patients from South-east Asia and shown to contribute to the hepatocarcinogenesis [24]–[27]. We therefore constructed HBx/50 and HBx/51 mutants for the study. Our results have demonstrated that liver cells with the full-length HBx and those with HBx mutants differentially responded to doxorubicin. Doxorubicin appears to have cytotoxicity towards HBx cells that contain the full-length HBx and CL cells that do not have HBx with the similar efficacy. However, HBx/51 cells that contain the HBx fragment of 51–154 a.a. were much more sensitive to doxorubicin whereas HBx50 cells that contain the HBx fragment of 1–50 a.a. were most resistant to the treatment. These findings indicate that in term of cell viability and growth, N-terminal HBx fragment of the first 1–50 a.a. may function differently from C-terminal HBx fragment of the last 51–154 a.a., with the former being promotive but the latter being inhibitory. Our data may explain contradictory results on HBx in liver cells as HBx has been reported to be both pro-apoptotic and anti-apoptotic [3]–[6]. The data are also supported by previous observations [25], [26], [28]. Oishi *et al.* have found that HBx fragment with 51–154 a.a., fails to exhibit colonigenic and tumorigenic abilities [28]. The mutants of HBx-truncated 27 or 35 a.a. at the C-terminal can strongly enhance the proliferation and growth of liver cells [25], [26]. Collectively, the cells containing the full length HBx are less sensitive towards doxorubicin-induced cell death than the cells containing HBx/51. This is likely due to fact that the full-length HBx contains the first 50 a.a. NH_2_ region that has the ability to resist cell death stimulation, as evident in this study. This first 50 a.a. region may block the function of the carboxy-terminus of HBx when the cells are stressed by anti-cancer drugs. Consequently, the full-length HBx was less sensitive than the HBx/51 in which the first 50 a.a. NH_2_ region has been omitted.

The acute or transient hypoxia is frequently shown to enhance the efficiency of anti-cancer treatments, mainly via facilitating apoptosis in cancer cells [9]–[12]. Our results support this concept since all 4 cell lines tested were much more sensitive to doxorubicin treatment in the hypoxic condition than in the normoxic condition. The ability of hypoxia to facilitate the cell death was demonstrated by the results of cell viability and TUNEL assays, as cell viability was decreased and the apoptosis was increased when the doxorubicin-treated cells were cultured in hypoxia compared with those in normoxia. The change in apoptotic molecules also supports the finding that hypoxia reduces the dose of doxorubicin. The pro-apoptotic molecule tBid was not detectable in cells treated with 2 µM of doxorubicin in normoxia and only detected in HBx/51 cells treated with 5 µM of doxorubicin after the culture of 48 h in normoxia. However, tBid was found in almost all cells treated with 2 µM of doxorubicin in hypoxia. It is important to note that the level of tBid is much higher in HBx/51 cells than in the other three cell lines treated with doxorubicin and hypoxia. To our best knowledge, the stimulation of Bid cleavage by hypoxia has not been reported in liver cells. Previous studies have indicated that the administration of tBid can inhibit the growth of HCC either *in vitro* or *in vivo* [18], [29], [30]. The results obtained by this study may suggest that the introduction of the acute hypoxic environment may further increase the efficiency of the treatment as Bid cleavage can be significantly promoted by hypoxia.

To investigate whether which pathway was responsible for the cleavage of Bid, the expression levels of Akt and p38 MAPK were analyzed since the activation of Bid is associated with Akt and p38 MAPK in certain cells [20]–[22]. Furthermore, in variety of human cancer cells, upregulation of p38 MAPK are known to function upstream of Bid to facilitate apoptosis [31], [32]. In our study, the cells treated with 5 µM doxorubicin under hypoxia showed a significant induction of tBid and activation of p38 MAPK but not Akt. In order to confirm the involvement of p38 MAPK, the p38 MAPK inhibitor SB203580 was introduced to investigate the role of p38 MAPK in the cleavage of Bid. The result showed that SB203580 was most effective in HBx cells that contain the full-length HBx as the tBid was undetectable and the cells were more proliferative when they were treated with 2 µM of doxorubicin under hypoxia. Different from the cells containing the full-length HBx or the first 50 a.a. HBx, SB203580 was less effective in the HBx/51 cells that contain the last 104 a.a., as HBx/51 cells had the lowest cell proliferation when they were treated with 2 µM of doxorubicin under hypoxia. Therefore these findings suggest that with respect to the recovery of the cell growth, the action of SB203580 is the least effective in cells containing C-terminal deletion of HBx. This finding is consistent with the concept that C-terminal truncated HBx mutants play a critical role in the hepatocarcinogenesis [24]–[27].

Our study has also shown that HBx enhanced the level of HIF-1α when cells were in the hypoxic condition, especially with a prolonged culture, but such an enhancement was not evident in the cells cultured in normoxia. HBx50 and HBx51 appeared not to have a significant effect on the expression of HIF-1α. Thus, HIF-1α may contribute little to the effects of HBx, HBx50 and HBx51 on cell growth and chemotherapy in the model tested. Our finding is somewhat in line with one previous study in which HBx increases HIF-1α in hypoxic 293 cells [33]. However, the effects of HBx mutant fragments between their study and ours are hard to compare since the fragments used in both studies are different. Moon *et al.* have demonstrated that HBx can increase the level of HIF-1α in 293 cells under both normoxic and hypoxic conditions [34]. The reason for the conflicting result is not clear but it may be due the different types of cells used.

In conclusion, HBx/51 cells, which contain the C-terminal HBx, are most sensitive towards doxorubicin especially when the cells are in the hypoxic condition, compared with the cells containing the full-length HBx or the N-terminal HBx. Hypoxia may help to produce tBid in the cells treated with doxorubicin, and eventually facilitate apoptosis. The activation of p38 MAPK, but not Akt, is involved in the cleavage of Bid in the cell model tested. In contrast to HBx/51 cells, HBx/50 cells, which contain the N-terminal HBx, are relatively resistant to doxorubicin. Therefore, the N-terminal domain and C-terminal domain of HBx function differentially towards doxorubicin. Nevertheless, in either case, hypoxia is a factor in favor of cell death.

## Supporting Information

Figure S1 Expression of HBx fragments in the established HBx, HBx/50 and HBx/51 cells. Total RNA was isolated and subjected to RT-PCR for relevant HBx fragments. The sequences of the primers for PCR were as follows. HBx: HBx_1F: 5′ – CCG AAT TCA ACC ATG GCT AGG CTG TGC – 3′ and HBx-154R: 5′ – GAA TGC GGC CGC ATT AGG CAG AGG TGA AAA AGT – 3′; HBx/50: HBx_1F: the same as described above for HBx and HBx_50R: 5′ – GAA TGC GGC CGC TTA CCC GTG GTC GGT CGG TAC – 3′; HBx/51: HBx_51F: 5′ – CCG AAT TCA CTA TGG CGC ACC TCT CTT TAC GCG – 3′ and HBx_154R: the same described above for HBx. PCR products were examined by running a 2% agarose gel. HBx HBx/50 and HBx/51 fragments/bands were detected at 478 bp, 176 bp and 336 bp respectively.
(TIF)
