# Supplementary figures and images for: Differential Functions of C- and N-Terminal Hepatitis B x Protein in Liver Cells Treated with Doxorubicin in Normoxic or Hypoxic Condition
Source: PLoS One. 2012 Nov 29;7(11):e50118. doi: 10.1371/journal.pone.0050118 (PMC3510201; doi:10.1371/journal.pone.0050118)

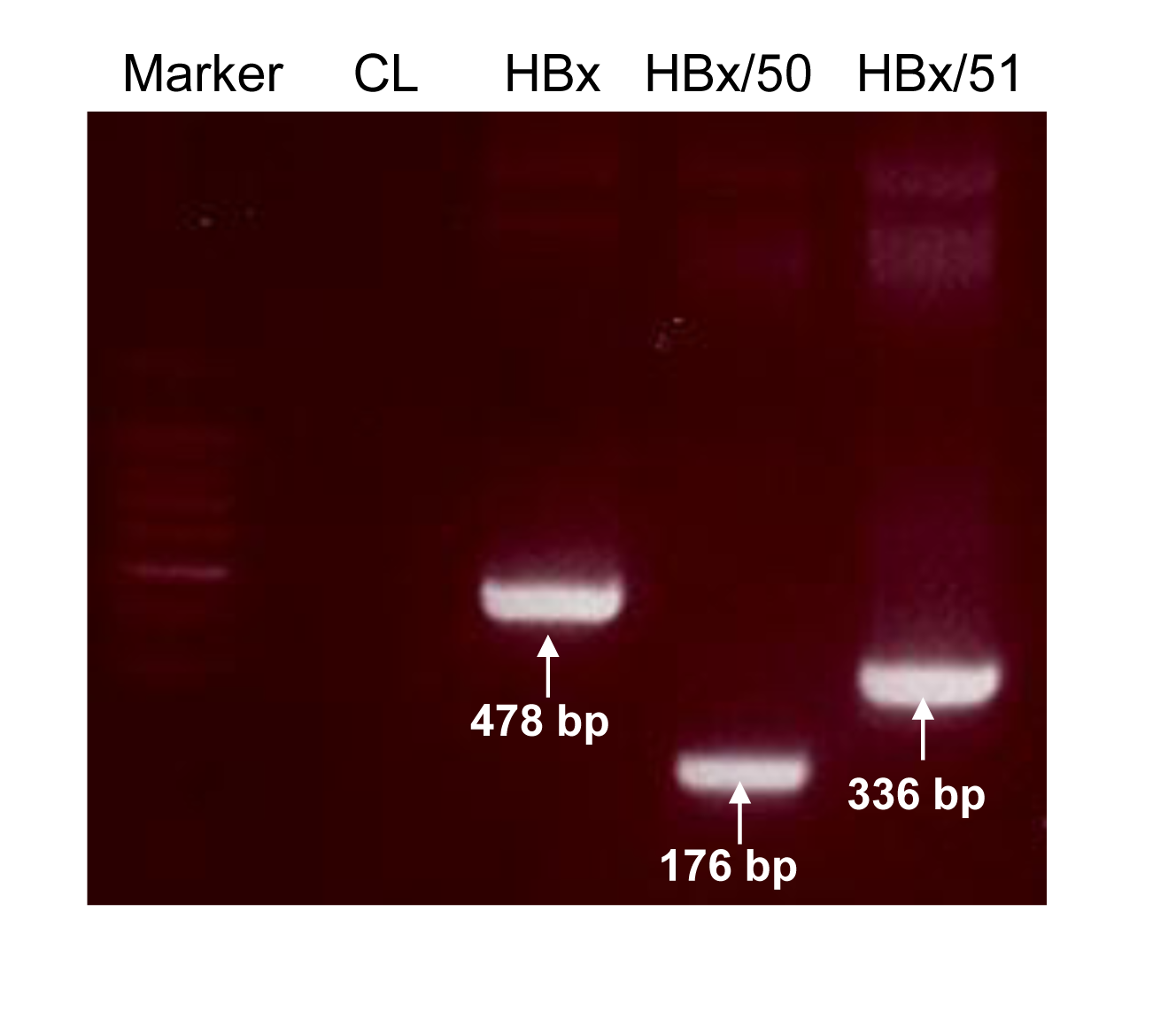

Supplement: Figure S1 — Expression of HBx fragments in the established HBx, HBx/50 and HBx/51 cells. Total RNA was isolated and subjected to RT-PCR for relevant HBx fragments. The sequences of the primers for PCR were as follows. HBx: HBx_1F: 5′ – CCG AAT TCA ACC ATG GCT AGG CTG TGC – 3′ and HBx-154R: 5′ – GAA TGC GGC CGC ATT AGG CAG AGG TGA AAA AGT – 3′; HBx/50: HBx_1F: the same as described above for HBx and HBx_50R: 5′ – GAA TGC GGC CGC TTA CCC GTG GTC GGT CGG TAC – 3′; HBx/51: HBx_51F: 5′ – CCG AAT TCA CTA TGG CGC ACC TCT CTT TAC GCG – 3′ and HBx_154R: the same described above for HBx. PCR products were examined by running a 2% agarose gel. HBx HBx/50 and HBx/51 fragments/bands were detected at 478 bp, 176 bp and 336 bp respectively. (TIF) [file pone.0050118.s001.tif]
